# Neurodevelopmental Delay Diagnosis Rates Are Increased in a Region with Aerial Pesticide Application

**DOI:** 10.3389/fped.2017.00116

**Published:** 2017-05-24

**Authors:** Steven D. Hicks, Ming Wang, Katherine Fry, Vignesh Doraiswamy, Eric M. Wohlford

**Affiliations:** ^1^Department of Pediatrics, State University of New York, Upstate Medical University, Syracuse, NY, United States; ^2^Department of Pediatrics, Milton S. Hershey Medical Center of Penn State University, Hershey, PA, United States; ^3^Department of Public Health Sciences, Milton S. Hershey Medical Center of Penn State University, Hershey, PA, United States; ^4^Department of Pediatrics, Palo Alto Medical Foundation, Palo Alto, CA, United States; ^5^Department of Pediatrics, University of California at San Francisco, San Francisco, CA, United States

**Keywords:** autism spectrum disorder, pyrethroid, neurodevelopmental disorder, pesticide, environment

## Abstract

A number of studies have implicated pesticides in childhood developmental delay (DD) and autism spectrum disorder (ASD). The influence of the route of pesticide exposure on neurodevelopmental delay is not well defined. To study this factor, we examined ASD/DD diagnoses rates in an area near our regional medical center that employs yearly aerial pyrethroid pesticide applications to combat mosquito-borne encephalitis. The aim of this study was to determine if areas with aerial pesticide exposure had higher rates of ASD/DD diagnoses. This regional study identified higher rates of ASD/DD diagnoses in an area with aerial pesticides application. Zip codes with aerial pyrethroid exposure were 37% more likely to have higher rates of ASD/DD (adjusted RR = 1.37, 95% CI = 1.06–1.78, *p* = 0.02). A Poisson regression model controlling for regional characteristics (poverty, pesticide use, population density, and distance to medical center), subject characteristics (race and sex), and local birth characteristics (prematurity, low birthweight, and birth rates) identified a significant relationship between aerial pesticide use and ASD/DD rates. The relationship between pesticide application and human neurodevelopment deserves additional study to develop safe and effective methods of mosquito prevention, particularly as communities develop plans for Zika virus control.

## Introduction

The recent emergence of the Zika virus and the discovery that perinatal Zika exposure increases fetal morbidity and mortality has led to a call for swift prevention measures (1). Among these measures is the expansion of aerial pesticide application to control the *Aedes aegypti* mosquito. Pesticides, which are designed to interfere with synaptic transmission in the central nervous system (2, 3), are indeed successful at reducing mosquito-borne illness. However, a growing number of studies suggest that the neurotoxic properties of pesticides may have unintended consequences in the brain of developing children (4–7). Although the instinct of communities is to protect children at all costs, it may be prudent to recall the Hippocratic Oath of physicians: “Primum non-nocere,” or “First, do no harm.”

The Childhood Autism Risks from Genetics and Environment study assessed the association of prenatal agricultural pesticide exposure and risk of autism spectrum disorder (ASD) or developmental delay (DD) in 970 children aged 2–5 years (6). The study found that children with ASD or DD were more likely than typically developing controls to have had organophosphate (OP), pyrethroid, or carbamate pesticides applied near their mother’s home, particularly during the third trimester of gestation. Although subsequent reviews have pointed out that such evidence does not establish causation, it does bolster the hypothesis that exposure to pesticides may contribute to neurodevelopmental disorders in genetically vulnerable children (8). Acetylcholine, a neurotransmitter that plays a critical role in learning, attention, and memory (9), is reduced in frontal and parietal cortices of children with ASD (10) and is a target of OP and pyrethroid pesticides (PPs) (11). This may represent one mechanism through which pesticides target the developing brain.

Pyrethroids are one specific class of pesticides that may increase the risk of ASD and DD (6, 12). In a study by Roberts and colleagues (4), maternal exposure to the pyrethroid bifenthrin during critical periods of gestation was associated with an increased risk of ASD diagnosis. In comparison, a recent study of 287 children showed that urinary pyrethroid levels at 6 years were negatively correlated with verbal comprehension and working memory, but maternal pyrethroid levels during gestation were not (7). These studies contribute to a growing body of evidence that pyrethroids can negatively affect neurodevelopment. The influence of exposure route and timing remain unclear. This question is an important one given that pyrethroid use has increased following a ban of OP pesticides in residential areas (13) and is a primary platform in Zika virus prevention.

Pyrethroid pesticides are employed in the central New York (NY, USA) county of Onondaga as a preventive tool against mosquitoes carrying eastern equine encephalitis (EEE) and West Nile virus (WNV). Each summer, the Onondaga County Department of Health supervises the aerial application (using aircraft) of 220 L (66 kg) of PP (Masterline^®^ Kontrol 30-30 Concentrate; Austin, TX, USA) to a four zip-code area collectively known as the Cicero Swamp. The effects of this application on neurodevelopmental patterns in local children have not been investigated.

The present study examines the prevalence of neurodevelopmental delay (rates of ASD and DD measured as the proportion of children diagnosed) in a central New York locale exposed to yearly aerial pyrethroid applications. The study tests the hypothesis that rates of regional ASD/DD diagnoses are related to route (aerial application) of PP exposure.

## Materials and Methods

### Subject Identification

This study employed an ecological cross-sectional design. Permission for a retrospective review of pediatric charts from outpatient electronic medical records at the State University of New York (SUNY) Upstate Medical University was obtained from the Institutional Review Board. On April 16, 2015, an electronic chart review was used to identify all children younger than 20 years evaluated during a 5-year period (March 15, 2010 to March 15, 2015) with ICD-9 diagnostic codes corresponding to ASD (F84.0) or DD (R62.50) at one of six pediatric outpatient clinics (psychiatry and behavioral sciences private practice; pediatric general provider clinic; psychiatry; pediatrics autism, behavior, and feeding program; pediatric child development center; or pediatric genetics). Neurodevelopmental characteristics, including developmental test scores [Vineland Adaptive Behavioral Scales; Autism Diagnostic Observation Schedule (ADOS); Baylee Scales of Infant and Toddler Development; and Developmental Assessment of Young Children (DAY-C)], genetic test results, psychiatric comorbidities [attention deficit hyperactivity disorder (ADHD) and anxiety], parental drug/cigarette use, age at diagnosis, and birth month were recorded for all subjects when available in the electronic medical record.

Subjects were divided by zip code of residence into aerial-exposed and control zip codes. Aerial-exposed zip codes included four zip codes directly exposed to aerial application of PPs by the Onondaga County Health Department EEE prevention program and four adjacent zip codes (all within 2.5 km of the aerial application area). The distance of 2.5 km was chosen based upon the study by Shelton et al. (6), which determined that children within this exposure distance had increased risk of ASD or DD. Sixteen surrounding zip codes greater than 2.5 km from the application area served as control regions. The control zip codes were not exempt from standard methods of pesticide application (e.g., hydraulic spraying, manual dispensing of granules, or controlled droplet application) by state-certified commercial applicators, but had no state-approved aerial applications.

### Population Demographics

Publicly available databases were employed to determine demographic data, perinatal data, and total pesticide exposure for each of the control and aerial-exposed zip codes. The total number of children younger than 20 years in each zip code was obtained from the 2013 American Community Survey (ACS).^1^ This database also provided the percentage of male children, the percentage of children below the poverty level, and the ethnic breakdown of children younger than 20 years in each zip code. The 2010–2012 New York State Vital Statistics Database^2^ was used to identify the number of births, the percentage of premature births (less than 37 weeks gestation), and the percentage low birthweight deliveries (less than 2,500 g) for each zip code over the specified 3-year period.

### Regional Pesticide Exposure

Pesticide exposure within each zip code was estimated using data from the New York State Department of Environmental Conservation (DEC) Database.^3^ This database is maintained in cooperation with Cornell University and uses standard quality assurance processes to correct and validate mandated submissions of pesticide use by commercial applicators. Average pesticide exposure (in kilograms) over a 3-year period (2007–2009) was determined for each zip code using the most recently available data on the DEC website. Pesticide exposure in kilograms per square kilometer was calculated for each zip code. Details regarding the timing and volume of aerial pyrethroid application over the exposed zip codes (application of 220L between late July and early September of each calendar year from 2003 to present) were provided by the Onondaga County Department of Health and are reflected in the totals on the DEC website.

### Prevalence of Neurodevelopmental Delay

The prevalence of neurodevelopmental delay (ASD and DD) for each zip code was calculated by dividing the number of observed cases at SUNY Upstate outpatient clinics by the total number of children residing in that zip code (as reported by the 2013 ACS). Comparisons were made between the 8 aerial-exposed zip codes and the 16 control zip codes. To validate the rates of ASD/DD diagnoses seen in control and aerial-exposed regions, the rates of ASD/DD in 30 additional zip codes in Central New York were recorded using electronic medical record review as described above. These zip codes had standard pesticide application procedures, but no aerial pesticide application practices. Total number of children and total pesticide application (kilograms) were recorded for these regions as well. Eight zip-code combinations were generated 360 times from this group, and mean rates of ASD/DD diagnoses were compared against the control and aerial-exposed zip-code rates.

### Regional Rates of Health Center Utilization

To prevent bias introduced by regional rates of health center utilization, we examined whether children from aerial-exposed zip codes were seen at SUNY Upstate outpatient clinics more often than children residing in control zip codes. The number of children from each region who were seen at our regional health center for four common pediatric visits were recorded: constipation, ADHD, asthma, and well-child checks. A retrospective chart review was used to identify subjects aged 20 years or older seen at the institution’s outpatient clinics during the same 5-year period (March 15, 2010 to March 15, 2015) with an ICD-9 diagnostic code corresponding to constipation (K59.00), ADHD (F90.9), asthma (J45.909), or well-child check (Z00.129). A health center utilization ratio was calculated using the total number of children with a specified diagnosis from the 8 aerial-exposed zip codes divided by the total number of children with that same diagnosis from the 16 control zip codes. These utilization ratios were then compared to the ratio seen for ASD/DD, as well as the ratio of the total number of children living in aerial-exposed and control zip codes.

### Statistics

Two-sample *t*-test or Wilcoxon rank-sum test was used for continuous variables, and Pearson’s chi-square test or Fisher’s exact test was used for categorical variables to identify between-group differences in demographic, perinatal, pesticide, and ASD prevalence data, as appropriate. Two-proportion *Z*-tests were used to examine health center utilization ratios for common pediatric diagnoses. A prevalence ratio for ASD/DD diagnosis was calculated for children living in the aerial-exposed zip codes compared with control zip codes. Pearson’s statistic was used to determine correlations between ASD/DD prevalence, aerial pesticide exposure, birth month, pesticide exposure per square kilometer, percent of premature births, percent of low birthweight births, race (% Caucasian), percent of children below the poverty level, and percent of male children for each zip code. A Poisson regression model was fitted to estimate the association between ASD/DD prevalence and aerial pyrethroid exposure (as a binary exposure variable) across zip codes adjusted to potential confounders including socioeconomic status (percent of children below the poverty level), incidence of premature births, number of births, percent of low birthweight births, distance to the tertiary referral center, race (percent of Caucasian children within the zip code), pesticide exposure per square kilometer, population density, and sex (percent of male children within the zip code). The overdispersion of the Poisson model was tested based on *z*-statistic, and the goodness-of-fit test was performed based on the residual deviance. The confounders that might influence pesticide exposure or ASD/DD prevalence were initially identified with Bayesian approach with a drected acyclic graph (Figure S2 in Supplementary Materials) and formally evaluated with stepwise regression procedures based on deviance chi-square tests. Factors without statistically significant impact remained in the final model due to projected clinical relevance. Statistical analyses were conducted using SAS version 9.4, and MetaboAnalyst 3.0 (14).

## Results

### Demographics

Demographic data from 8 aerial-exposed zip codes were compared with 16 control zip codes (Table 1). There were no significant differences between aerial-exposed and control zip codes in land area (*p* = 0.92), number of children younger than 20 years (*p* = 0.59), premature births (*p* = 0.47), low birthweight births (0.24), children with white race (*p* = 0.82), children below the poverty level (*p* = 0.14), or child sex (*p* = 0.63). There was an increased rate of neonatal death per 1,000 births in the control zip codes (*p* = 0.02).

**Table 1:** **Demographic data from 8 zip codes exposed to aerial pyrethroid pesticides and 16 control zip codes**.

	Zip code	Land Area (sq km)	Children <20 years	Premature births (per 100 births)	Low birthweight (per 100 births)	Neonate death rate (per 1,000 births)	White (%)	Below poverty level (%)	Male (%)
Aerial-exposed zip codes	1	36	5,000	49 (10)	32 (7)	2.0	4,715 (94)	165 (3)	2,568 (51)
	2	21	743	7 (8)	5 (6)	0.0	739 (99)	79 (11)	371 (50)
	3	38	872	12 (12)	8 (8)	0.0	851 (98)	110 (13)	435 (50)
	4	45	3,319	38 (10)	24 (6)	5.0	3,067 (92)	262 (8)	1,733 (52)
	5	8	1,942	29 (11)	12 (5)	0.0	1,746 (90)	410 (21)	1,022 (53)
	6	17	4,174	65 (10)	42 (7)	4.7	3,907 (94)	351 (8)	2,140 (51)
	7	66	2,268	20 (8)	14 (5)	0.0	2,252 (99)	399 (18)	1,140 (50)
	8	3	755	11 (9)	13 (10)	0.0	744 (99)	38 (5)	375 (50)

	**Sum**	**233**	**19,073**	**231**	**150**		**18,021**	**1,813**	**9,783**
	**Mean**	**29**	**2,384**	**29**	**19**	**1.5**	**2,253**	**227**	**1,223**

Control zip codes	9	28	2,082	30 (11)	15 (5)	3.6	1,959 (94)	283 (14)	1,073 (52)
	10	5	4,354	87 (13)	69 (10)	7.4	2,308 (53)	1,924 (44)	1,994 (46)
	11	29	2,959	38 (9)	32 (8)	4.8	2,784 (94)	145 (5)	1,529 (52)
	12	25	587	7 (11)	6 (9)	0.0	576 (98)	66 (11)	300 (51)
	13	6	3,769	77 (11)	73 (11)	4.3	2,880 (76)	1,165 (31)	1,911 (51)
	14	6	2,762	18 (9)	14 (7)	14.0	2,138 (77)	351 (13)	1,374 (50)
	15	38	750	12 (10)	11 (9)	0.0	706 (94)	158 (21)	381 (51)
	16	79	2,306	20 (8)	20 (8)	0.0	2,260 (98)	383 (17)	1,157 (50)
	17	55	923	10 (11)	7 (8)	10.8	889 (96)	157 (17)	471 (51)
	18	24	7,984	94 (9)	68 (6)	1.8	7,106 (89)	798 (10)	4,074 (51)
	19	63	2,526	21 (10)	15 (7)	14.2	2,104 (83)	58 (2)	1,331 (53)
	20	8	3,349	42 (9)	26 (6)	4.2	3,238 (97)	258 (8)	1,674 (50)
	21	24	2,858	25 (10)	18 (7)	0.0	2,689 (94)	66 (2)	1,470 (51)
	22	13	3,975	64 (9)	44 (6)	7.2	3,629 (91)	537 (14)	2,023 (51)
	23	28	603	5 (4)	5 (4)	8.8	425 (71)	136 (23)	298 (50)
	24	20	2910	31 (7)	20 (5)	2.3	2,765 (95)	469 (16)	1,454 (50)

	**Sum**	**451**	**44,697**	**581**	**444**		**38,455**	**6953**	**22,514**
	**Mean**	**28**	**2,794**	**36**	**28**	**5.2**	**2,403**	**435**	**1,407**

*p* Value		0.92	0.59	0.47	0.24	0.02	0.82	0.14	0.63

*The 2013 American Community Survey was used to determine the number of children younger than 20 years, the percentage of children of Caucasian (white) descent, the percentage of children below the poverty level, and the percentage of male children in each zip code. The 2010–2012 New York State Vital Statistics data from March 2014 was used to determine the rate of premature births, rate of low weight (<2 kg) births, and the neonatal death rate*.

### Health Center Utilization Rates

There were less children living in the 8 aerial-exposed zip codes (*n* = 19,073) compared with the 16 control zip codes (*n* = 44,697). The ratio of children younger than 20 years in aerial-exposed zip codes compared with control zip codes was 0.43 (Table 2). In comparison, lower ratios of children were seen at our health center from aerial-exposed zip codes for common pediatric diagnoses: constipation (*Z* = −2.84), ADHD (*Z* = −3.60), asthma (*Z* = −4.26), and well-child checks (*Z* = −14.36). Despite reduced utilization of our health center by aerial-exposed zip regions, the utilization ratio for ASD/DD diagnoses by these regions exceeded the population ratio (*Z* = 2.29; *p* = 0.02).

**Table 2:** **Health center utilization rates for common pediatric conditions**.

Diagnosis	Children (#) aerial pyrethroid pesticide (PP)	Children (#) control	Prevalence ratio	*Z*-score	*p* -Value
Autism spectrum disorder (ASD)/developmental delay (DD)	159	298	0.53	2.29	0.022
Constipation	238	689	0.35	−2.84	0.004
Attention deficit hyperactivity disorder	57	226	0.25	−3.60	0.001
Asthma	228	735	0.31	−4.26	0.000
Well child	343	1805	0.19	−14.4	0.000
Total children	19,073	44,697	0.43		

*The number of children with each diagnosis is shown for zip codes with aerial PP exposure and control zip codes. Diagnoses were identified by review of electronic medical records from our regional medical center. Note that control zip codes have a higher rate of health center utilization for all common diagnoses except ASD and DD*.

### Pesticide Exposure

**Figure 1:**
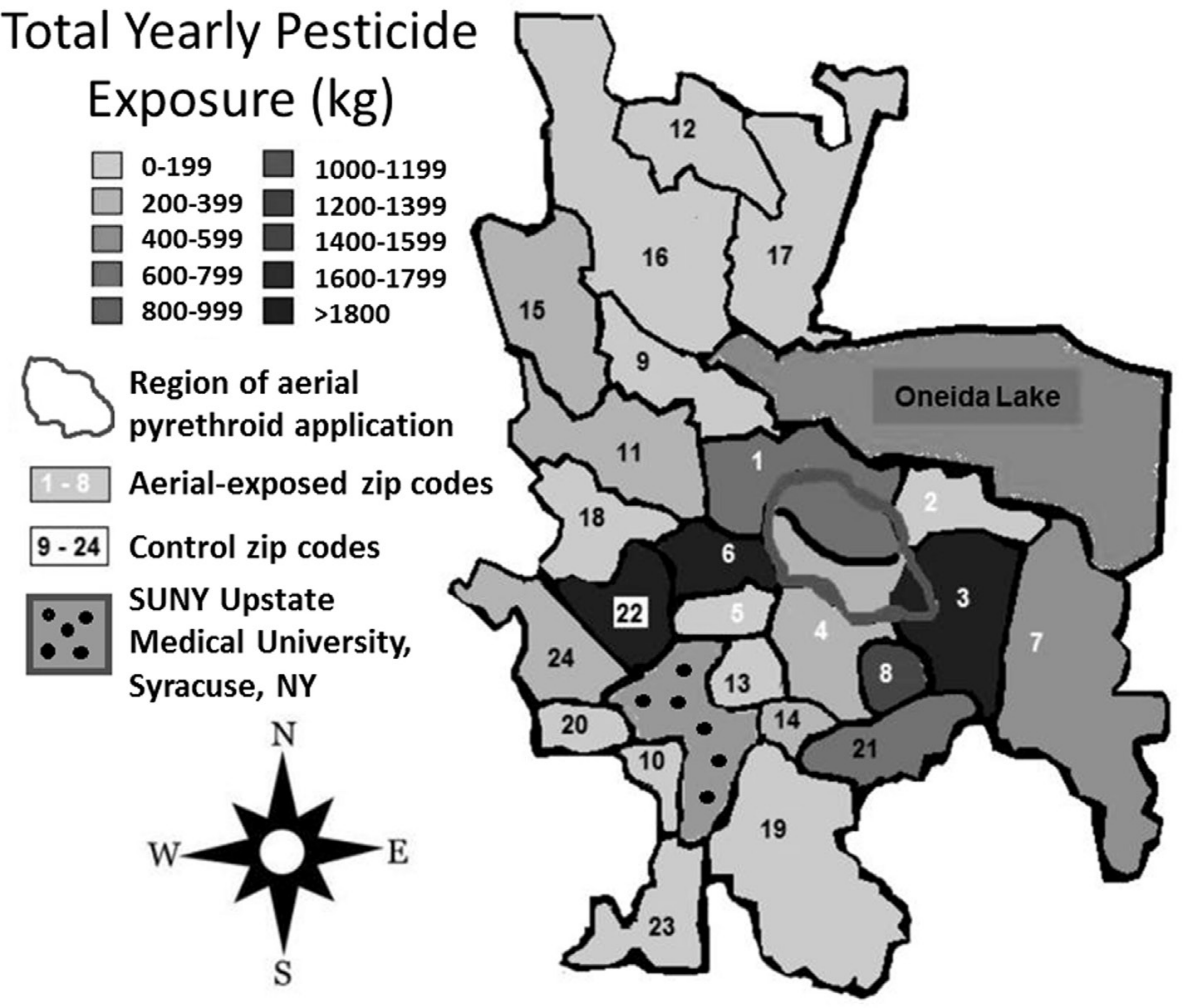
**Regional pesticide exposure**. Mean yearly pesticide exposure for each zip code is shown in kilograms. These levels were determined using the most recently available data from the New York State Department of Environmental Conservation Mandated Reporting Database. Total pesticide exposure includes pyrethroids, organophosphates, and other pesticide families regardless of class or application method employed. Aerial-exposed zip codes are numbered 1–8 in white, and control zip codes are numbered 9–24 in black. Note that there was no statistical difference between mean concentration of pesticide exposure between aerial-exposed zip codes (49 kg/km^2^) and control zip codes (9 kg/km^2^).

Between 2007 and 2009, the average yearly pesticide burden for the eight aerial-exposed zip codes was 11,299 kg (Table 3). This was a higher burden than the 4,164 kg of pesticide exposure seen across the 16 control zip codes (*p* = 0.02). Concentration of pesticide exposure for the 8 aerial-exposed zip codes (86 kg/km^2^) was not significantly higher (*p* = 0.20) than that in the 16 control zip codes (15 kg/km^2^). These data included the aerial application of 220 L of PPs over the aerial-exposed zip codes each year. A geographical representation of the aerial application zone along with mean yearly pesticide exposures for each zip code is displayed in Figure 1.

**Table 3:** **Average yearly pesticide burden for control and aerial-exposed zip codes**.

	Zip code	Pesticide (kg)	Pesticide (kg/km^2^)
Aerial-exposed zip codes	1	1,207	33
	2	27	1
	3	5,184	136
	4	376	8
	5	25	3
	6	1,817	107
	7	1,530	23
	8	1,133	378

	**Sum**	**11,299**	**690**
	**Mean**	**1,412**	**86**

Control zip codes	9	2	0
	10	0	0
	11	340	12
	12	16	1
	13	3	1
	14	0	0
	15	252	7
	16	52	1
	17	65	1
	18	141	6
	19	197	3
	20	13	2
	21	700	29
	22	1,967	153
	23	36	1
	24	382	19

	**Sum**	**4,164**	**233**
	**Mean**	**260**	**15**

*p* Value		0.02	0.20

*Pesticide exposure is expressed here as mean yearly weight exposure in kilograms from 2007 to 2009. There was significantly higher total pesticide exposure in the aerial-exposed zip codes, but not a higher pesticide concentration in kilograms per square kilometer*.

**Figure 2:**
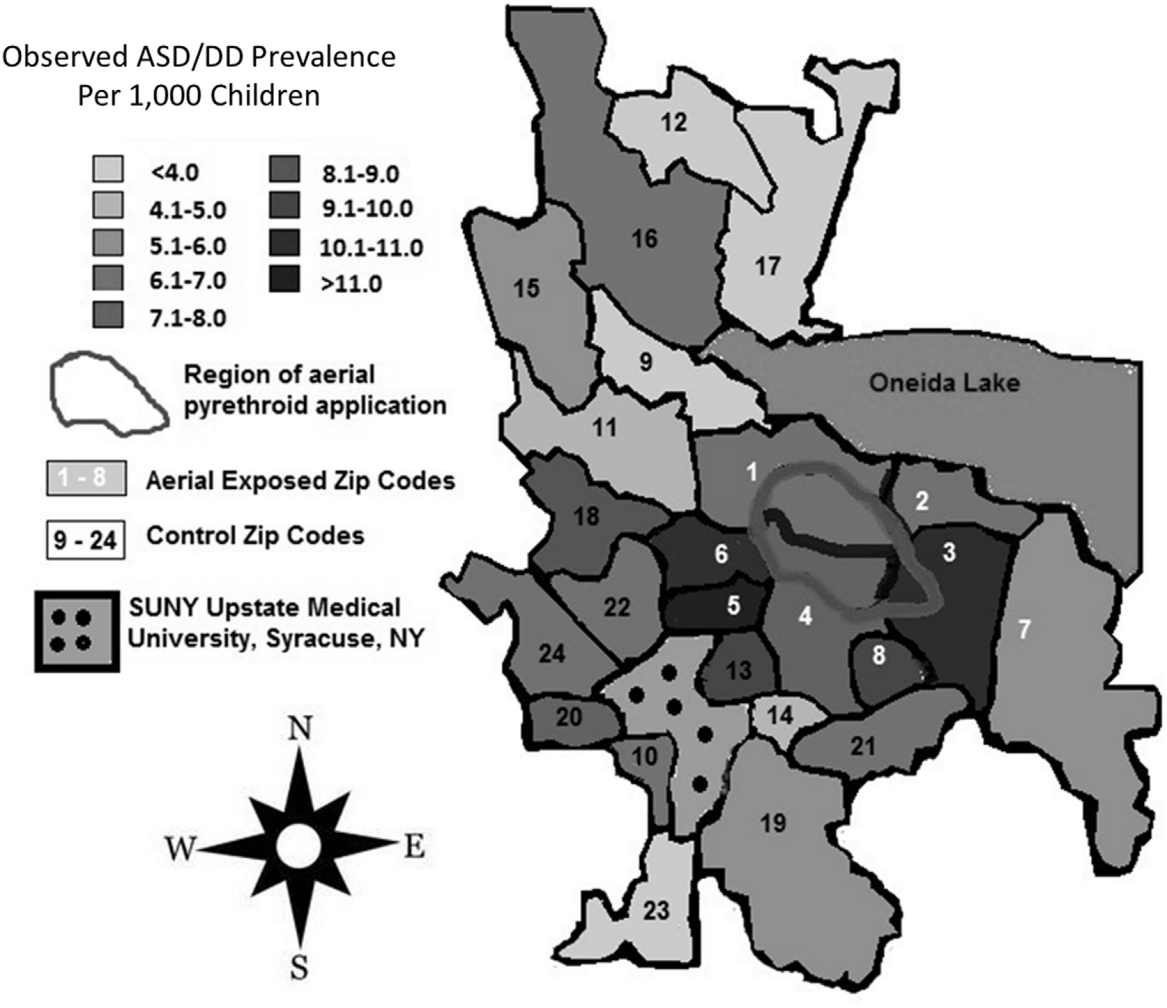
**Regional rates of autism spectrum disorder (ASD) and developmental delay (DD) diagnoses**. Total numbers of children diagnosed with ASD/DD in each zip code were identified with a retrospective review of records at the State University of New York (SUNY) Upstate Regional Medical Center, and prevalence rates were calculated using publicly available American Community Survey data for each zip code. Zip codes exposed to aerial pyrethroid pesticides had a higher prevalence of ASD/DD than control zip codes with standard pesticide application methods.

A review of SUNY Upstate electronic medical records revealed 159 children from aerial-exposed zip codes with a visit diagnosis of ASD or DD between March 15, 2010, and March 15, 2015 (Table 4). Of those 159 children, 121 (76%) were on the autistic spectrum. There were 251 children seen at SUNY Upstate with a diagnosis of ASD or DD from the 16 control zip codes during this same period. Of those children, 197 (78%) were on the autistic spectrum. These figures yield mean observed ASD/DD diagnosis rates of 1 of 115 (0.0087) in the aerial-exposed zip codes compared with 1 of 196 (0.0051) in the control zip codes. A geographical representation of the observed ASD/DD diagnosis rate for aerial-exposed and control zip codes is displayed in Figure 2. The adjusted prevalence ratio for ASD/DD diagnosis in the children exposed to aerial pyrethroids was 1.37 (95% CI: 1.06–1.78; *p* = 0.02) compared with the children living in the control zip codes.

**Table 4:** **Prevalence of autism spectrum disorder (ASD) or developmental delay (DD) diagnosis at the regional medical center for children from aerial-exposed and control zip codes**.

	Zip code	# ASD and DD	ASD/DD prevalence
Aerial-exposed zip codes	1	33	0.0066
	2	5	0.0067
	3	9	0.0103
	4	26	0.0078
	5	22	0.0113
	6	43	0.0103
	7	13	0.0057
	8	8	0.0106

	**Sum**	**159**	
	**Mean**	**20**	**0.0087**

Control zip codes	9	5	0.0024
	10	14	0.0032
	11	13	0.0044
	12	2	0.0034
	13	29	0.0077
	14	11	0.0040
	15	3	0.0040
	16	9	0.0039
	17	3	0.0033
	18	62	0.0078
	19	12	0.0048
	20	24	0.0072
	21	22	0.0077
	22	22	0.0055
	23	4	0.0066
	24	16	0.0055

	**Sum**	**251**	
	**Mean**	**19**	**0.0051**

*p* Value		0.51	0.0003

*There was a higher mean prevalence of ASD/DD diagnosis in aerial-exposed zip codes (1/115, 0.0087) compared with control zip codes (1/196, 0.0051)*.

A cross-validation procedure was used to compare the rate of ASD/DD diagnoses in the aerial-exposed zip codes (0.0087) against ASD/DD diagnosis rates at our medical center from 30 additional zip codes in Central New York without aerial pesticide application. Random eight zip code combinations were generated 360 times from this group (Table S1 in Supplementary Material) and resulted in zero examples of prevalence exceeding that observed in the aerial-exposed zip codes (*p* < 0.001). Of the 360 combinations, 115 had ASD/DD diagnoses rates that exceeded that seen in the original control group (*p* = 0.32). Note that the mean total pesticide exposure in these 30 zip codes (1,689 kg) did not differ from that seen aerial-exposed regions (1,412 kg; *p* = 0.76).

Pearson correlation testing (Figure S1 in Supplementary Material) revealed positive correlations between aerial-exposure and ASD/DD prevalence (*r* = 0.58), as well as total pesticide exposure (in kilograms per kilometer) and ASD/DD prevalence (*r* = 0.41). There was an inverse correlation between ASD/DD prevalence and distance to the medical center (*r* = −0.45), as well as neonatal death rate per 1,000 births (*r* = −0.43). There was no correlation between birth month and ASD/DD prevalence (*r* = −0.034).

A Poisson regression model controlling for regional poverty (% children below poverty line), race (% Caucasian), sex (% male), birth characteristics (e.g., prematurity, low birthweight, and birth rate), population density (children per square kilometer), distance from the medical center (kilometer), and total pesticide exposure (kilograms per square kilometer) identified a significant relationship between aerial pesticide exposure and ASD/DD rates (Table 5). The analysis demonstrated a significant association between aerial pyrethroid exposure and ASD/DD prevalence (adjusted RR = 1.37; *p* = 0.02).

**Table 5:** **Poisson regression analysis controlling for clinically relevant, confounding variables to estimate the direct effect of aerial pyrethroid exposure on ASD/DD prevalence**.

	Estimate	SE	Adjusted RR	95% CI for adjusted RR	*p* Value
Intercept	−7.550	1.380	0.0005	3.52–0.01	<0.001
Aerial pyrethroid exposure (±)	0.316	0.133	1.37	1.06–1.78	0.02
Births per year	0.0005	0.0002	1.00	1.00–1.00	0.02
Premature births (%)	0.026	0.056	1.03	0.92–1.15	0.64
Low birthweight (%)	0.048	0.048	1.05	0.95–1.15	0.32
Caucasian (%)	0.022	0.010	1.02	1.00–1.04	0.03
Below poverty level (%)	0.009	0.009	1.01	0.99–1.03	0.28
Male (%)	0.0001	0.011	1.00	0.98–1.02	0.99
Pesticide (kg/km^2^)	−0.0001	0.001	1.00	1.00–1.00	0.86
Distance to medical center (km)	−0.039	0.020	0.96	0.92–1.00	0.05
Population density (children/km^2^)	−0.0004	0.001	1.00	1.00–1.00	0.95

*ASD, autism spectrum disorder; DD, developmental delay; CI, confidence interval*.

Clinical characteristics for the ASD/DD subjects in both aerial-exposed and control zip codes are displayed in Table 6. There was no difference observed in child age, sex, age at ASD/DD diagnosis, or birth month between the two groups. No differences were noted in parental nicotine/alcohol use, and the groups had no difference in comorbid anxiety or ADHD. There were no difference between the two groups in percentage of children with ASD and no difference in those with known genetic disorders. In those children with developmental testing, no differences were detected in mean ADOS, DAY-C, or BSID scores. Composite adaptive behavior as measured by Vineland Scales was nominally higher in children from the aerial-exposed group, but this difference did not reach statistical significance (*p* = 0.06).

**Table 6:** **Clinical characteristics of subjects with ASD and DD from aerial-exposed and control zip codes**.

Measurement	Aerial-exposed zip codes	Control zip codes	*p*-Value
Mean age (years)	9.0	9.1	0.91
Female (%)	25.8	20.0	0.18
Mean age at Dx (months)	65	68	0.74
ASD (%)	76	78	0.25
ADOS score	10	12	0.31
BSITD score	79	65	0.12
DAY-C score	85	83	0.69
Vineland score	78	66	0.06
Comorbid attention deficit hyperactivity disorder (%)	31.4	24.1	0.10
Comorbid anxiety (%)	15.7	21.7	0.14
Parental nicotine/EtOH use (%)	13.8	13.6	0.94
Fall birth (%)	28.3	36.0	0.14
Syndromic disorder (%)	10.7	9.6	0.73

*There were no differences observed between groups in developmental characteristics. Fall births in the months between September and December were measured to assess the percentage of children in the target zip codes who would have received third trimester exposure to aerial application of pyrethroid pesticides. Children with syndromic disorders represent all children with a known genetic condition (including trisomies, chromosomal deletions), or diagnosed syndrome (e.g., neurofibromatosis, fetal alcohol spectrum disorder, tuberous sclerosis, Down syndrome, and Rhett syndrome)*.

*ASD, autism spectrum disorder; DD, developmental delay; Dx, diagnosis; ADOS, Autism Diagnostic Observation Schedule; BSITD, Baylee Scales of Infant and Toddler Development; DAY-C, Developmental Assessment of Young Children; EtOH, ethanol*.

## Discussion

This study examined the prevalence of ASD and DD in a central New York region with unique aerial exposure to the PP Masterline^®^ Kontrol. When compared with surrounding areas, the zip codes exposed to yearly aerial pyrethroid spraying had a higher prevalence of ASD/DD. Total pesticide exposure (kilograms per square kilometer) was correlated with ASD/DD prevalence, but did not contribute significantly to a Poisson regression model of the data. There was also no correlation between birth month (i.e., timing of gestational pyrethroid exposure) and ASD/DD prevalence.

Although this study is observational and does not establish a causal relationship between pyrethroid exposure and ASD/DD, it adds to a growing body of literature that shows a relationship between pesticides and ASD/DD (4, 6, 7). To our knowledge, this is the first study to show a relationship between the route of pesticide exposure and rates of neurodevelopmental delay. It raises intriguing questions about the safety of pesticide use in our society and how the manner in which those pesticides are applied might affect brain development in children.

Why might the route of pesticide exposure be so important? Each summer, the Onondaga County Health Department in Upstate New York combats EEE and WNV through aerial application of Kontrol between July and September. Prior to this application, residents are advised to (1) stay indoors for up to 1 h following spraying and keep windows closed; (2) close outdoor vents of window-unit air conditioners; (3) remove outdoor children’s toys, outdoor furniture, and clotheslines; and (4) cover gardens or rinse homegrown produce with water prior to consumption (15). It is unknown what percent of residents receive and fully comply with these recommendations. There are obvious technical difficulties involved with removing outdoor playground equipment or covering large gardens. These areas represent potential exposure sites for pregnant mothers or young children in early stages of neurodevelopment. Pesticide levels in the urine or blood of children from these areas have not been specifically studied in areas with aerial pesticide application.

A recent study of cortical neurons *in vitro* found that pesticide exposure caused specific transcriptional changes that mimic expression changes in the autistic human brain (16). Although transcriptome patterns across the groups of this study are unknown, there was no difference between the percentage of children with known genetic syndromes in aerial-exposed and control zip codes. There was also no difference between measures of autistic (ADOS), adaptive (Vineland), or social (DAY-C) behaviors. Future studies employing molecular sequencing analyses could be beneficial in identifying unique transcriptional profiles in children with ASD/DD exposed to PPs.

The recent emergence of Zika virus and associated cases of microcephaly may be relevant when considering the findings presented in this study. Zip codes employing aerial pesticides did so to combat WNV, another member of the flavivirus family. Although WNV is not known to cause overt dysmorphic features (17), its influence on long-term developmental outcomes is not well studied (18). Like Zika virus, WNV causes symptoms in less than 25% of infected individuals and less than 1% present with meningitis (19). Therefore, it is possible that children in zip codes with aerial pesticide exposure also experience increased rates of *in utero* exposure to WNV. Neither head circumferences nor WNV antibody titers have been studied from children in this region.

There are a number of difficulties in assessing the association between environmental exposures and neurodevelopmental outcomes. One of the primary difficulties involves accurate identification of children with ASD/DD within a defined geographic area (20). In the current study, that task has been accomplished through a review of records from a major tertiary referral center. The benefits of this approach are that SUNY Upstate is the only regional children’s hospital and the only academic medical center in an 80 mile radius, which includes a catchment area of approximately 1.8 million people. Children are diagnosed with ASD and DD at Upstate by regional experts in pediatrics, psychiatry, and childhood development using Diagnostic and Statistical Manual of Mental Disorders criteria. The limitation to this approach is that a number of children with ASD and DD may be managed by outlying primary pediatricians without specialist referral and are likely missed. This may explain why the average prevalence of ASD/DD in this study (0.0087 or 1/115) is roughly half that reported nationally (0.022 or 1/45) at the time of the records review. Rates of childhood constipation and asthma reported in this study (~1/67) are also one-third of the rates reported nationally (1/20). Thus, our data medical center likely provides care for one-third of children in the study region, and this is represented by consistent subsampling across diagnoses in the study data.

To increase the identification of ASD and DD in the community, records from schools and outlying evaluation centers could be employed. However, this approach carries its own set of limitations, such as reliance on diagnostic reporting by non-medical professionals (i.e., teachers and parents). To demonstrate that our sampling of subjects is an accurate snapshot of the region, we have provided a measure of regional health center utilization. The number of children in the exposed zip codes is 43% of the population of children in the control zip codes. Yet the number of children from aerial-exposed zip codes seen at SUNY Upstate Medical University for well-child checks and common pediatric diagnoses (asthma, constipation, ADHD) is consistently 20–30% of that seen from control zip codes. This suggests that diagnosis rates for ASD/DD may even underestimate the number of children in aerial-exposed zip codes with neurodevelopmental delay. The Poisson regression model employed in this study shows that the distance of each zip code to SUNY Upstate Medical Center does influence observed prevalence rates of ASD/DD. Even when accounting for this influence, the impact of aerial pyrethroid exposure remains a significant variable in relation to ASD/DD prevalence.

It should also be noted that this study was not designed to detect the influence of exposure timing on ASD/DD risk. The study was limited by availability of data resources, which restricted chronological overlap of birth statistics, population demographics, pesticide application, and ASD/DD diagnosis rates. The most up-to-date information for each respective resource was used in this study. In addition, we do not have information on where mothers resided during pregnancy, and this is a limitation of the current study. We observed no difference in ASD/DD diagnosis rates for children with birthdays between September and December (i.e., presumed third trimester exposure to July to August aerial pesticide spraying) relative to children born in other months of the year. The question of pesticide exposure timing is important and has been previously investigated by Shelton et al. (6).

A final consideration in studies of this nature is the principle of ecological fallacy or the use of group data to draw conclusions about individual health characteristics. While these results show an association between aerial pesticide exposure and rates of ASD/DD diagnosis at a regional health center, they do not support the conclusion that individual risk of autism increases with pesticide exposure. Furthermore, the application of these findings on a broader scale (statewide or nationwide) cannot be assessed without a broader multicenter study.

Aerial application of PPs has been an important public health tool in combating WNV and EEE virus. The results of this study suggest that this practice may have influences on pediatric neurodevelopment. Further studies are necessary to clarify the relationship between pesticide exposure and neurodevelopmental outcomes. It will be important to directly assess the biochemical burden of pesticide metabolites in the urine of children with ASD/DD and compare the epigenetic profiles of these children to those without pesticide exposure. In addition, knowledge about maternal and fetal exposure to flaviviruses such as WNV (assessed through antibody detection) could be critical in evaluating factors associated with ASD/DD risk in these groups. This information would help communities make decisions about the safety and utility of aerial pesticide application.

## Ethics Statement

Permission for a retrospective review of the electronic medical records at the State University of New York (SUNY) Upstate Medical University was obtained from that institution’s Institutional Review Board. Not applicable. The study examined neurodevelopmental delay diagnoses in a pediatric population, but consent was not required for review of records. All collected health information was deidentified.

## Author Contributions

SH conceived the study, collected and analyzed the data, and drafted the manuscript. EW contributed to study design and data collection. KF was involved in study design. VD contributed to manuscript preparation. MW participated in statistical analysis and manuscript preparation.

## Conflict of Interest Statement

The authors declare that the research was conducted in the absence of any commercial or financial relationships that could be construed as a potential conflict of interest. The reviewer, DS, and the handling editor declared their shared affiliation, and the handling editor states that the process nevertheless met the standards of a fair and objective review.

## Footnotes

^1^http://www.factfinder.census.gov.

^2^https://www.health.ny.gov/statistics/chac/perinatal/.

^3^http://magritte.psur.cornell.edu/09report/Sales_zip_srch.htm.
