# Supplementary material for: Neurodevelopmental Delay Diagnosis Rates Are Increased in a Region with Aerial Pesticide Application
Source: Front Pediatr. 2017 May 24;5:116. doi: 10.3389/fped.2017.00116 (PMC5443159; doi:10.3389/fped.2017.00116)
Supplement: Supplementary file 1 [file image_1.pdf]

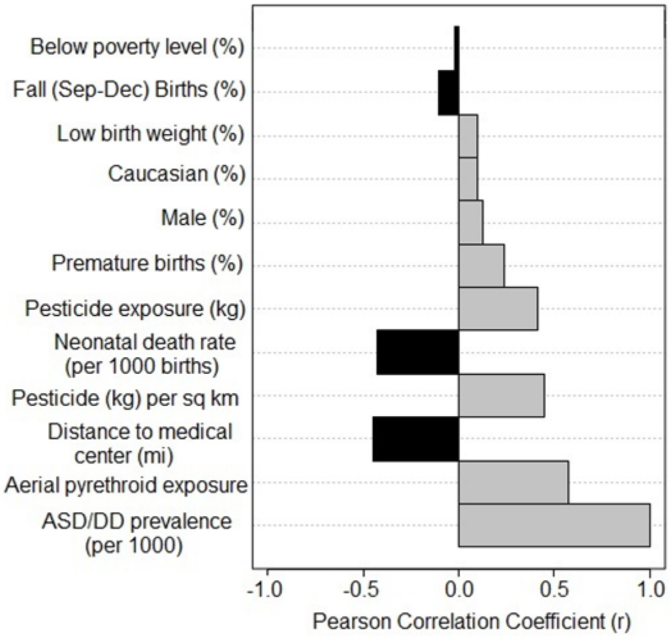

Supplementary Figure 1. Factors correlating with prevalence of autism spectrum disorder (ASD) and developmental delay (DD). The presence of aerial pyrethroid exposure was most strongly correlated with zip code ASD/DD prevalence. Note that zip code distance to the medical center in miles (mi) was more strongly correlated with ASD/DD prevalence (r = -0.45) than total pesticide exposure in kilograms (kg) per square kilometer (r = 0.41). There was no correlation (r = -0.034) between percent of children born in the fall and ASD/DD prevalence, a rough estimate of third trimester to summer aerial pesticide spraying.
