# Supplementary material for: Neurodevelopmental Delay Diagnosis Rates Are Increased in a Region with Aerial Pesticide Application
Source: Front Pediatr. 2017 May 24;5:116. doi: 10.3389/fped.2017.00116 (PMC5443159; doi:10.3389/fped.2017.00116)
Supplement: Supplementary file 2 [file image_2.pdf]

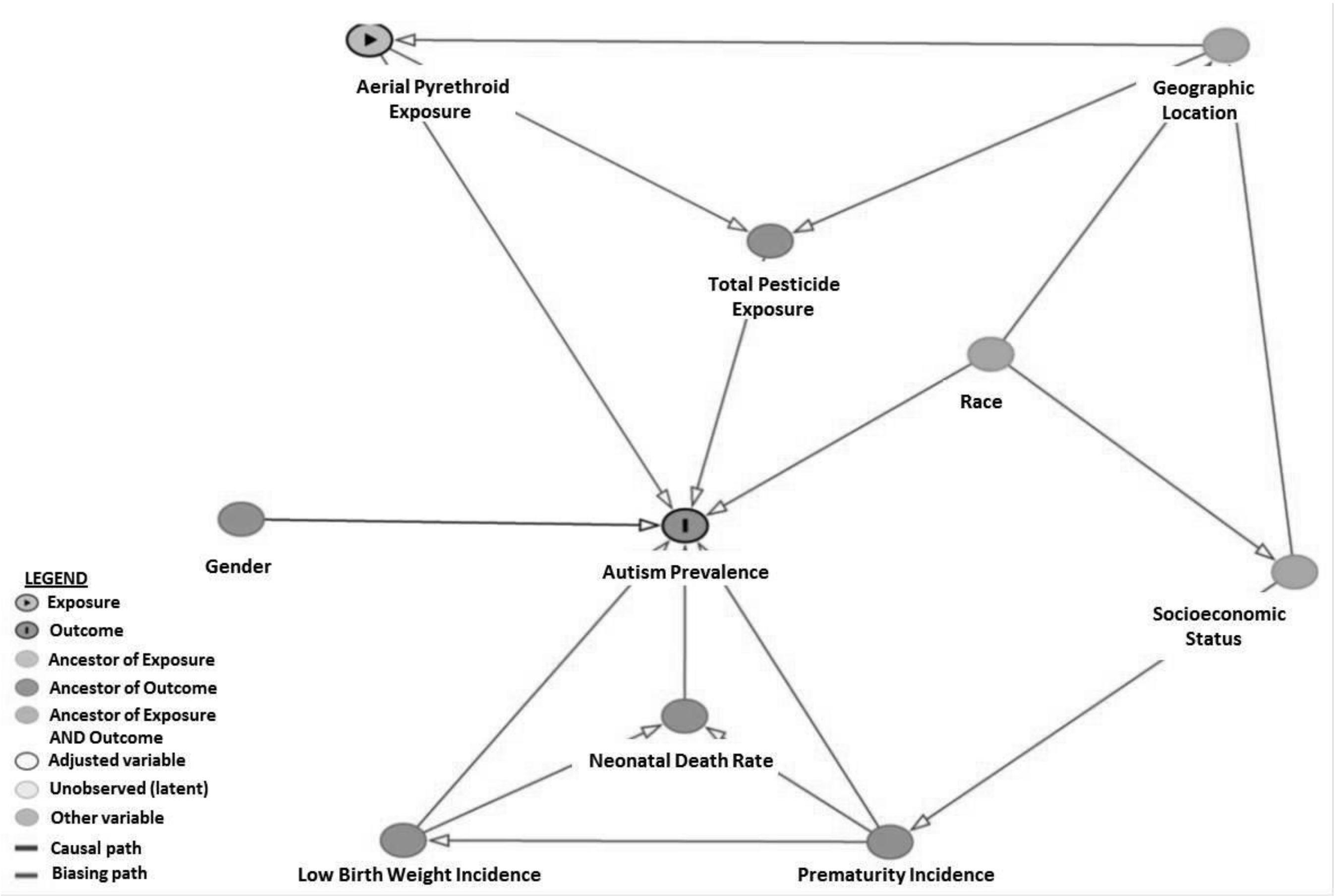

Supplementary Figure 2. Directed Acyclic Graph evaluating the change in estimate of autism prevalence based on potential confounding variables. The model implies the following conditional independences: 1) Aerial pyrethroids $\perp$ Gender, 2) Aerial pyrethroids $\perp$ Low Birth Weight Incidence $|$ Socioeconomic Status, 3) Aerial pyrethroids $\perp$ Low Birth Weight Incidence $|$ Prematurity Incidence, 4) Aerial pyrethroids $\perp$ Low Birth Weight Incidence $|$ Geographic Location, 5) Aerial pyrethroids $\perp$ Prematurity Incidence $|$ Geographic Location, 6) Aerial pyrethroids $\perp$ Race $|$ Geographic Location, 7) Aerial pyrethroids $\perp$ Socioeconomic Status $|$ Geographic Location, 8) Aerial pyrethroids $\perp$ Neonatal Death Rate $|$ Prematurity Incidence. Based upon this model the minimal sufficient adjustment sets for estimating the direct effect of aerial pesticide exposure on autism prevalence include: 1) Geographic location, total pesticide exposure; 2) Prematurity incidence, race, total pesticide exposure; 3) Race, socioeconomic status, total pesticide exposure. These sets were used to inform a linear regression model of the data.
