# Supplementary material for: Neurodevelopmental Delay Diagnosis Rates Are Increased in a Region with Aerial Pesticide Application
Source: Front Pediatr. 2017 May 24;5:116. doi: 10.3389/fped.2017.00116 (PMC5443159; doi:10.3389/fped.2017.00116)
Supplement: Supplementary file 3 [file table_1.docx]

Supplementary Table 1. Cross validation of autism/developmental delay (ASD/DD) diagnoses rates. A list of 30 Central New York zip codes was generated regardless of total pesticide exposure or zip code demographics. Rates of ASD/DD diagnoses at our regional medical center were determined through review of the electronic medical record. Random eight zip code combinations were generated 360 times from this group of control regions and ASD/DD prevalence rates were calculated (color scaled with highest rates shown in red). None of the combinations yielded an average prevalence of ASD/DD diagnosis that exceeded the rate observed in the 8 aerial-exposed zip codes (p < 0.001) and 115/360 combination yielded diagnoses rates higher than the control zip codes (p=0.3).
